# Differential Effects of Male and Female Gonadal Hormones on the Intrathymic T cell Maturation

**DOI:** 10.1155/2001/52789

**Published:** 2001

**Authors:** Branka Pejčić-Karapetrović, Duško Kosec, Gordana Leposavić

**Affiliations:** 1 lnstitute for Immunology and Virology “Torlak” Immunology Research Center “Branislav Janković” 458 Vojvode Stepe Belgrade 11221 Serbia and Montenegro; 2 Faculty of Pharmacy 450 Vojvode Stepe Belgrade 11221 Serbia and Montenegro

**Keywords:** adult rats, thymus size, thymus cellularity, thymocyte maturation, gonadectomy, sexual dimorphism

## Abstract

The study was undertaken to further elucidate a role of gonadal hormones in maintenance of normal thymocyte maturation and sexual dimorphism in the intrathymic T-cell development. Rats of both sexes were gonadectomized or sham-gonadectomized (controls) at age of 2 and 6 months, and 30 days later the thymus size, cellularity and thymocyte composition were evaluated. In both control and gonadectomized rats, in spite of age, sexual dimorphism in the thymus size and cellularity was found. Gonadectomy in 2-month-old rats of both sexes increased the thymus cellularity, volumes of both cortex and medulla and thymus size (to a less extent in males), while in 6-month-old rats, in this respect, it was effective only in females. In ovariectomized (OVX) rats the increase in volume of cortex was more marked in younger rats, while that of medulla did not differ between rats of different age. It seems obvious that in both groups of OVX rats the volume of medullary non-lymphoid component was enlarged (the increase in medullary volume was more pronounced than that in its cellularity). Unlikely, in rats orchidectomized (ORX) at age of 2 months the volume of this component was either decreased or unaltered (the increase in the volume of medulla was less conspicuous than that in the number of medullary thymocytes). In control and gonadectomized rats of both ages, sexual dimorphism in the composition of thymocyte subsets was also observed. Gonadectomy in 2-month-old rats affected distinct stages of thymocyte maturation in male (increased the relative proportions of CD4+8+TCRαβ^low^ cells and their CD4–8+TCRαβ^low^ precursors and decreased those of the most mature CD4+8-TCRαβ^high^ and CD4–8+TCRαβ^high^ cells) and female rats (decreased only the percentage of the least mature CD4–8-TCRαβ-cells). In older rats only ovariectomy had impact on the relative proportion of thymocytes decreasing, besides the relative proportion of CD4–8-TCRαβ^-^ cells, those of CD4–8+TCRαβ^-^, CD4–8+TCRαβ^low^, positively selected CD4+8+TCRαβ^high^ and the most mature CD4+8-TCRαβ^high^, CD4–8+TCRαβ^high^ cells and exerting an opposite effect on the percentages of CD4+8+TCRαβ^-^ and CD4+8+TCRαβ^low^ cells. Thus, results showed sex- and age-dependent changes in sensitivity of both the developing thymocytes and non-lymphoid cells to long-lasting gonadal deprivation.


*Developmental Immunology*, 2001, Vol. 8(3-4), pp. 305-317
Reprints available directly from the publisher
Photocopying permitted by license only

© 2001 OPA (Overseas Publishers Association)
N.V. Published by license under
the Harwood Academic Publishers imprint,
par of The Gordon and Breach Publishing Group,
a member of the Taylor and Francis Group

### Differential Effects of Male and Female Gonadal Hormones on the Intrathymic T cell Maturation

BRANKA PEJČIĆ-KARAPETROVIĆ[a], DUŠKO KOSEC[a] and GORDANA LEPOSAVIĆ[ab]*

[a]*Institute for Immunology and Virology "Torlak", Immunology Research Center "Branislav Janković", 458 Vojvode Stepe and* [b]*Faculty of Pharmacy, 450 Vojvode Stepe, 11221 Belgrade, Yugoslavia*

The study was undertaken to further elucidate a role of gonadal hormones in maintenance of normal thymocyte maturation and sexual dimorphism in the intrathymic T-cell development. Rats of both sexes were gonadectomized or sham-gonadectomized (controls) at age of 2 and 6 months, and 30 days later the thymus size, cellularity and thymocyte composition were evaluated. In both control and gonadectomized rats, in spite of age, sexual dimorphism in the thymus size and cellularity was found. Gonadectomy in 2-month-old rats of both sexes increased the thymus cellularity, volumes of both cortex and medulla and thymus size (to a less extent in males), while in 6-month-old rats, in this respect, it was effective only in females. In ovariectomized (OVX) rats the increase in volume of cortex was more marked in younger rats, while that of medulla did not differ between rats of different age. It seems obvious that in both groups of OVX rats the volume of medullary non-lymphoid component was enlarged (the increase in medullary volume was more pronounced than that in its cellularity). Unlikely, in rats orchidectomized (ORX) at age of 2 months the volume of this component was either decreased or unaltered (the increase in the volume of medulla was less conspicuous than that in the number of medullary thymocytes). In control and gonadectomized rats of both ages, sexual dimorphism in the composition of thymocyte subsets was also observed. Gonadectomy in 2-month-old rats affected distinct stages of thymocyte maturation in male (increased the relative proportions of CD4+8+TCR$\alpha\beta^{low}$ cells and their CD4–8+TCR$\alpha\beta^{low}$ precursors and decreased those of the most mature CD4+8-TCR$\alpha\beta^{high}$ and CD4–8+TCR$\alpha\beta^{high}$ cells) and female rats (decreased only the percentage of the least mature CD4–8-TCR$\alpha\beta$-cells). In older rats only ovariectomy had impact on the relative proportion of thymocytes decreasing, besides the relative proportion of CD4–8-TCR$\alpha\beta^{-}$ cells, those of CD4–8+TCR$\alpha\beta^{-}$, CD4–8+TCR$\alpha\beta^{low}$, positively selected CD4+8+TCR$\alpha\beta^{high}$ and the most mature CD4+8-TCR$\alpha\beta^{high}$, CD4–8+TCR$\alpha\beta^{high}$ cells and exerting an opposite effect on the percentages of CD4+8+TCR$\alpha\beta^{-}$ and CD4+8+TCR$\alpha\beta^{low}$ cells. Thus, results showed sex- and age-dependent changes in sensitivity of both the developing thymocytes and non-lymphoid cells to long-lasting gonadal deprivation.

*Keywords:* adult rats, thymus size, thymus cellularity, thymocyte maturation, gonadectomy, sexual dimorphism

* Address for correspondence: Professor Gordana Leposavić, M.D., Ph.D., Institute for Immunology and Virology "Torlak", Immunology Research Center "Branislav Janković", 458 Vojvode Stepe, 11221 Belgrade, YUGOSLAVIA. Tel/Fax: 381 11 467 465, E-mail:leposa@afrodita.rcub.bg.ac.yu

### INTRODUCTION

Clinical and experimental observations clearly indicate that both cell-mediated and humoral immune responses are more effective in females than in males (Grossman, 1984, 1985, 1990; Ansar-Ahmed et al., 1985; Kovacs and Olsen, 1998). Although the underlying mechanisms of this sexual dimorphism have not been understood, yet, it has been suggested that immune-endocrine (particularly gonadal) interactions produce a significant contribution to the development and maintenance of this phenomenon (Grossman, 1990; Kovacs and Olsen, 1998). A variety of data showed that gonadal steroid hormones modulate the skin allograft rejection time in experimental animals (Graff, 1969), as well as susceptibility and expression of autoimmune diseases in both experimental models (Roubinian et al., 1977a, 1978, 1979; Steinberg et al., 1980; Pozzilli et al., 1993; Vidal et al., 1994) and humans (Agnello et al., 1983; Bizzaro et al., 1987; Ben-Chetrit and Ben-Chetrit, 1994). Similarly to humans, most models of autoimmune disease in animals occur preferentially or with greater severity in females than males (Grossman, 1990; Kovacs and Olsen, 1998). The classic description of this sexual dimorphism was made in the NZB/W murine lupus model. Disease prevalence and the rate of progression to mortality are greater in females than in males of this F1 hybrid strain (Steinberg et al., 1980). Gonadectomy of NZB/W male mice results in a mortality curve that is equivalent to intact females. Androgen treatment in female rats of this strain causes almost complete disappearance of disease manifestation (Roubinian et al., 1977a, 1978, 1979). Conversely, estradiol administration to gonadectomized female rats enhances disease progression (Roubinian et al, 1978). Since removal of the thymus prevents the amelioration of disease evoked by androgens, it has been suggested that the thymus represents the key point within the pathways of interaction between male steroid hormones and the immune system (Roubinian et al., 1977b). Moreover, it has been assumed that the immunological dimorphism establishes early in the course of thymocyte maturation and that the concentration of gonadal steroid hormones is the major factor in its development and maintenance (Grossman, 1985). To support this hypothesis are data showing that both thymocytes and thymus epithelial cells (TEC) express receptors for estrogens and androgens (Grossman et al., 1979; Gulino et al., 1985; Seiki et al., 1988; Kawashima et al., 1991; Kawashima et al., 1992; Viselli et al., 1995; Kohen et al., 1998), and that TEC also bear the receptors for progesterone (Fujii-Hanamoto et al., 1985; Kawashima et al., 1991). In the same line are our previous data showing sexual dimorphism in the composition of thymocyte subsets delineated either by expression of CD4/CD8 coreceptor molecules or TCRαβ (Leposavić et al., 1995, 1996). Moreover, our previous study suggested that the gonadal steroids are more important for the development than for the maintenance of sexual dimorphism in the thymocyte composition. To test this assumption and to further elucidate the modulatory role of sex steroids in intrathymic T-cell maturation in adult rats of both sexes and of different age, we assessed the effects of one month gonadal deprivation upon the thymus structure and composition of thymocyte subsets. The rats were gonadectomized at age of 2 and 6 months, as it has been shown that in rats major developmental changes in the thymus structural and thymocyte phenotypic characteristics occur during the first month of life and from six month onward (Quaglino et al., 1998). Since the process of intrathymic T-cell development is commonly depicted as a linear flow between successively more differentiated compartments, thus that thymocytes at different developmental stages are situated within distinct parts of the thymus (Scollay et al., 1984), in these rats the volumes of different thymus compartments and their cellularity were estimated. In addition, as the changes in cell surface molecules (TCRαβ and coreceptor CD4 and CD8 molecules) expression allow delineation and monitoring of the thymocyte populations at different stages of maturation, and therefore insight into the process of differentiation of T-lymphocyte precursors into functionally mature cells (Petrie et al., 1990; Tsuchida et al., 1994), the expression of these molecules on the thymocytes of the same animals was also analyzed.

TABLE I Thymus weight and total number of thymocytes, volumes of the cortex and medulla, and numbers of cortical and medullary thymocytes in rats sham-orchidectomized (sham-ORX), ORX, sham-ovariectomized (sham-OVX) and OVX at age of 2 months

| | *Sham-ORX* | *ORX* | *Sham-OVX* | *OVX* |
|---|---|---|---|---|
| THYMUS | | | | |
| Thymus weight (g) | $0.50 \pm 0.01^{**a}$ | $0.79 \pm 0.02^{**b,*c}$ | $0.26 \pm 0.02$ | $0.69 \pm 0.06^{**d}$ |
| Total number of thymocytes ($\times 10^7$) | $46.62 \pm 2\ 55^{**a}$ | $78.78 \pm 1.08^{**b,*c}$ | $22.83 \pm 0.71$ | $66.80 \pm 6.52^{**d}$ |
| CORTEX | | | | |
| Volume ($cm^3$) | $0.34 \pm 0.01^{**a}$ | $0.55 \pm 0.03^{**b}$ | $0.18 \pm 0.003$ | $0.53 \pm 0.06^{**d}$ |
| Number of thymocytes ($\times 10^7$) | $39.14 \pm 1.88^{**a}$ | $66.52 \pm 4.16^{**b}$ | $19.00 \pm 0.25$ | $59.57 \pm 6.81^{**d}$ |
| MEDULLA | | | | |
| Volume ($cm^3$) | $0.14 \pm 0.01^{**a}$ | $0.21 \pm 0.02^{**b,c}$ | $0.06 \pm 0.01$ | $0.12 \pm 0.009^{*d}$ |
| Number of thymocytcs ($\times 10^7$) | $7.48 \pm 0.89^{*a}$ | $12.26 \pm 0.97^{*b,c}$ | $3.83 \pm 0.73$ | $7.23 \pm 1.18^{*d}$ |

The results are expressed as mean ± SEM (n=5–7).
$^{*}$ $p \leq 0.05$; $^{**}$ $p \leq 0.01$.
[a] Sham-ORX vs. Sham-OVX.
[b] ORX vs. Sham-ORX.
[c] ORX vs. OVX.
[d] OVX vs. Sham-OVX.

### RESULTS

### Thymus Weight and Volumes of the Thymus Compartments

#### *Two-month-old rats*

In rats sham-gonadectomized at age of 2 months the volumes of both thymus cortex and medulla, and consequently the thymus weight, were significantly greater in males than in their female counterparts. Gonadectomy, at that age, in rats of both sexes significantly ($p<0.01$) increased the volumes of both thymus compartments, and thus ($p<0.01$) the thymus weight, as well. Ovariectomy was more effective than orchidectomy in increasing not only the cortical (the mean value was 194 % greater in OVX than in sham-OVX rats, and only 62 % greater in ORX than in sham-ORX animals), but also medullary volume (the mean value was 100 % greater in OVX than in sham-OVX rats and only 50 % grater in ORX than in sham-ORX rats), thus that the volume of cortical compartment in OVX rats was indistinguishable from that in ORX rats, while the volume of thymus medulla did not reach that in ORX rats. Although ovariectomy was more effective than orchidectomy in increasing the thymus weight (the mean value was 165 % greater in OVX than in sham-OVX rats and only 58 % greater in ORX than in sham-ORX animals), the thymus weight remained significantly ($p<0.05$) greater in male gonadectomized rats (Table I).

#### *Six-month-old rats*

In rats sham-operated at age of 6 months the volumes of both thymus compartments, and therefore the organ weight, were also significantly ($p<0.05$) greater in male than in female rats. Ovariectomy, but not orchidectomy, evoked a significant enlargement of the volumes of both thymus cortex (the mean value was 127 % greater in OVX compared with sham-OVX rats) and medulla (the mean value was 100 % greater in OVX compared with sham-OVX

rats), thus that the volume of thymus cortex was significantly ($p<0.05$) greater in OVX rats than that in ORX rats, while the volume of medulla did not significantly differ between these two groups of rats (Table II). Since only ovariectomy significantly ($p<0.01$) increased the thymus weight (the mean value was 120 % greater in OVX than that in sham-OVX rats), the thymus weight in OVX rats was significantly ($p<0.05$) greater than in ORX rats (Table II).

### Total Number of Thymocytes and Number of Thymocytes in the Thymus Compartments

### *Two-month-old rats*

In rats subjected to sham-gonadectomy at age of 2 months the numbers of thymocytes in both thymus compartments, and consequently the total number of lymphoid cells in thymus were significantly ($p<0.01$) higher in male than in female rats. One month after gonadectomy in rats of both sexes the numbers of thymocytes in thymus cortex (the mean value was 192 % higher in OVX than in sham-OVX rats and only 70 % higher in ORX than in sham-ORX rats), and medulla (the mean value was 89 % higher in OVX than in sham-OVX rats, and 64 % higher in ORX than in sham-ORX rats) were increased, thus that in the cortex of OVX rats the number of thymocytes attained that in the cortex of ORX rats, while the number of these cells in medulla remained significantly ($p<0.05$) greater in male rats than in their female counterparts. Although ovariectomy produced a more marked increase in the total thymocyte number (the mean value was 193 % higher in OVX than in sham-OVX rats and only 69 % higher in ORX than in sham-ORX rats), the total number of these cells remained still significantly ($p<0.05$) greater in male compared with their female counterparts (Table I).

TABLE II Thymus weight and total number of thymocytes, volumes of the cortex and medulla, and numbers of cortical and medullary thymocytes in rats sham-orchidectomized (sham-ORX), ORX, sham-ovariectomized (slam-OVX) and OVX at age of 6 months

| | *Sham-ORX* | *ORX* | *Sham-OVX* | *OVX* |
|---|---|---|---|---|
| THYMUS | | | | |
| Thymus weight (g) | $0.31 \pm 0.03^{*a}$ | $0.32 \pm 0.04^{*b}$ | $0.20 \pm 0.02$ | $0.44 \pm 0.05^{**c}$ |
| Total number of thymocytes ($\times 10^7$) | $28.47 \pm 2.22^{*a}$ | $28.73 \pm 3.34^{*b}$ | $19.23 \pm 1.87$ | $40.15 \pm 4.59^{**c}$ |
| CORTEX | | | | |
| Volume ($cm^3$) | $0.21 \pm 0.02^{*a}$ | $0.21 \pm 0.03^{*b}$ | $0.15 \pm 0.01$ | $0.34 \pm 0.05^{**c}$ |
| Number of thymocytes ($\times 10^7$) | $24.30 \pm 1.40^{*a}$ | $23.13 \pm 2.77^{*b}$ | $16.67 \pm 1.41$ | $36.18 \pm 4.56^{**c}$ |
| MEDULLA | | | | |
| Volume ($cm^3$) | $0.08 \pm 0.03^{*a}$ | $0.09 \pm 0.009$ | $0.04 \pm 0.006$ | $0.08 \pm 0.005^{*c}$ |
| Number of thymocytes ($\times 10^7$) | $4.17 \pm 0.84^{*a}$ | $5.60 \pm 0.95$ | $2.57 \pm 0.48$ | $3.98 \pm 0.49^{*c}$ |

The results are expressed as mean ± SEM (n=5–7).

$^{*}$ $p \leq 0.05$; $^{**}$ $p \leq 0.01$.

[a] Sham-ORX vs. Sham-OVX.

[b] ORX vs. OVX.

[c] OVX vs. Sham-OVX.

### *Six-month-old rats*

The total numbers of thymocytes in both cortex and medulla, and therefore in the whole organ were significantly ($p<0.05$) higher in male than in female rats sham-gonadectomized at age of 6 months. Orchidectomy at same age affected the total number of thymocytes in none of the thymus compartments, while ovariectomy evoked a significant increase in the number of thymocytes in both thymus compartments (the value of mean thymocyte number in cortex was 117 % and that in medulla was 55% higher in OVX than in sham-OVX rats), thus that in gonadectomized rats the total number of thymocytes was higher in the thymus cortex of females, while there was no significant difference between male and female rats in the number of these cells in medulla. Since gonadectomy significantly ($p<0.01$) affected the total number of thymocytes only in females (the mean value was 109 % higher in OVX that in sham-OVX), the total number of these cells was significantly ($p<0.05$) higher in gonadectomized female rats than in their male counterparts (Table II).

### Analysis of the Expression of CD4/CD8/TCRαβ

In respect to the expression of CD4 and CD8 molecules and that of TCRαβ (three levels of TCRαβ expression were distinguished: negative, low and high) we delineated and estimated relative proportion of twelve subsets of thymocytes (Tsuchida et al., 1994).

### *Two month-old rats*

In the male sham-gonadectomized rats the relative proportions of all subsets of thymocytes with undetectable level of TCRαβ (TCRαβ$^-$) except that of CD4+8- single positive (SP) cells were significantly lower compared with female controls. Orchidectomy affected the relative proportion of none of four subsets of TCRαβ$^-$ cells, while ovariectomy caused only a significant ($p<0.01$) decrease in the relative proportion of CD4–8- double negative (DN) thymocytes, but its value remained still greater than that in ORX rats, thus that in gonadectomized rats the relative proportions of all subsets of TCRαβ$^-$ thymocytes except that of CD4+8- SP were also significantly lower in male than in female rats (Table III).

TABLE III Percentage (%) of thymocytes within each of twelve subpopulations defined by the expression of CD4, CD8 and TCRαβ molecules in rats sham-orchidectomized (sham-ORX), ORX, sham-ovariectomized (sham-OVX) and OVX at age of 2 months

| *Thymocyte subpopulations* | *Sham -ORX (mean ± SEM)* | *ORX (mean ± SEM)* | *Sham-OVX (mean ± SEM)* | *OVX (mean ± SEM)* |
|---|---|---|---|---|
| CD4–8-TCRαβ- | 0.3 ± 0.04$^{**a}$ | 0.3 ± 0.03$^{**b}$ | 1.3 ± 0.05 | 0.9 ± 0.03$^{**c}$ |
| CD4+8-TCRαβ- | 0.4 ± 0.10 | 0.3 ± 0.3 | 0.3 ± 0.02 | 0.3 ± 0.03 |
| CD4–8+TCRαβ- | 0.7 ± 0.25$^{**a}$ | 0.8 ± 0.03$^{**b}$ | 1.8 ± 0.23 | 1.7 ± 0.01 |
| CD4+8+TCRαβ- | 35 ± 1.43$^{*a}$ | 36.4 ± 1.12$^{*b}$ | 37.6 ± 0.30 | 38.6 ± 0.26 |
| CD4–8-TCRαβ$^{low}$ | 0.7 ± 0.15$^{*a}$ | 0.8 ± 0.07$^{*b}$ | 0.5± 0.04 | 0.6 ± 0.03 |
| CD4+8-TCRαβ$^{low}$ | 0.9 ± 0.09$^{**a}$ | 0.9 ± 0.09$^{**b}$ | 0.4 ± 0.04 | 0.6 ± 0.03 |
| CD4–8+ TCRαβ$^{low}$ | 1.1 ± 0.5 | 1.8 ± 0.02$^{*d,**b}$ | 1.0 ± 0.07 | 1.1 ± 0.05 |
| CD4+8+TCRαβ$^{low}$ | 35.6 ± 1.46$^{*a}$ | 38.9 ± 0.75$^{**d}$ | 38.2 ± 0.28 | 38.5 ± 0.27 |
| CD4–8-TCRαβ$^{high}$ | 0.7 ± 0.19$^{*a}$ | 0.6 ± 0.02$^{**b}$ | 1.0 ± 0.04 | 1.1 ± 0.08 |
| CD4+8-TCRαβ$^{high}$ | 11.5 ± 1.61$^{**a}$ | 8.6 ± 0.31$^{**d}$ | 8.3 ± 0.1 | 8.0 ± 0.24 |
| CD4–8+TCRαβ$^{high}$ | 5.5 ± 1.19$^{**a}$ | 3.8 ± 0.34$^{*d}$ | 3.2 ± 0.09 | 3.1 ± 0.15 |
| CD4+8+TCRαβ$^{high}$ | 7.6 ± 1.19 | 7.0 ± 0.41 | 6.5 ± 0.07 | 5.6 ± 0.11 |

The results are expressed as mean ± SEM (n=5–7).
* p≤0.05; ** p≤0.01.
[a] sham-ORX vs. sham-OVX.
[b] ORX vs. OVX.
[c] OVX vs. sham-OVX.
[d] ORX vs. sham-OVX.

The analysis of relative proportions of thymocytes expressing low level of TCRαβ expression (TCRαβ$^{low}$) showed that the relative proportions of CD4–8- DN and CD4+8- SP cells were significantly higher, while that of CD4+8+ double positive (DP) cells was significantly (p<0.05) lower in sham-ORX than in sham-OVX rats. Orchidectomy significantly increased the percentage of CD4–8+ SP and CD4+8+ DP thymocytes expressing TCRαβ at low density, while ovariectomy did not affect the relative proportion of any of subsets of TCRαβ$^{low}$ cells, thus that in gonadectomized rats the relative proportions of all subsets of TCRαβ$^{low}$ thymocytes except that of CD4+8+ DP cells were higher in male than in female rats (Table III).

The relative proportions of both the subsets of SP cells (CD4+8- and CD4–8+) with high level of TCRαβ expression (TCRαβ$^{high}$) were significantly (p<0.01) higher, while that of CD4–8- (DN) cells was significantly (p<0.05) lower in sham-ORX compared with sham-OVX rats. Orchidectomy significantly decreased the relative proportions of both subsets of SP cells, while ovariectomy affected percentage of none of subsets of TCRαβ$^{high}$ cells, thus that in gonadectomized rats only the percentage of CD4–8-TCRαβ$^{high}$ cells remained significantly different in male compared with female rats (Table III).

### *Six month-old rats*

Relative proportion of none of the subsets of TCRαβ$^-$ thymocytes significantly differed between male and female rats sham-gonadectomized at age of six months. Orchidectomy affected relative proportion of none of the subsets of TCRαβ$^-$ cells, while ovariectomy significantly decreased the percentages of CD4–8-TCRαβ$^-$ and CD4–8+TCRαβ$^-$ cells and slightly, but significantly (p<0.01), increased the relative proportion of CD4+8+TCRαβ$^-$ cells. One month after gonadectomy the relative proportion of CD4–8+TCRαβ$^-$ thymocytes was significantly (p<0.01) higher, while that of CD4+8+TCRαβ$^-$ cells was significanly (p<0.01) lower in male than in female rats (Table IV).

TABLE IV Percentage (%) of thymocytes within each of twelve subpopulations defined by the expression of CD4, CD8 and TCRαβ molecules in rats sham-orchidectomized (sham-ORX), ORX, sham-ovariectomized (sham-OVX) and OVX at age of 6 monthsp

| *Thymocyte subpopulations* | *Sham-ORX (mean ± SEM)* | *ORX (mean ± SEM)* | *Sham-OVX (mean ± SEM)* | *OVX (mean ± SEM)* |
|---|---|---|---|---|
| CD4–8-TCRαβ- | 0.3 ± 0.04 | 0.5 ± 0.2 | 0.4 ± 0.01 | 0.3 ± 0.02$^{*a}$ |
| CD4+8-TCRαβ- | 0.4 ± 0.10 | 0.5 ± 0.11 | 0.4 ± 0.01 | 0.5 ± 0.08 |
| CD4–8+TCRαβ- | 1.1 ± 0.02 | 1.1 ± 0.07$^{**b}$ | 1.3 ± 0.05 | 0.7 ± 0.04$^{**a}$ |
| CD4+8+TCRαβ- | 37.9 ± 0.36 | 37.9 ± 0.39$^{**b}$ | 38.2 ± 0.10 | 40.0 ± 0.24$^{**a}$ |
| CD4–8-TCRαβ$^{low}$ | 0.5 ± 0.01 | 0.6 ± 0.10 | 0.8 ± 0.06 | 0.8 ± 0.02 |
| CD4+8-TCRαβ$^{low}$ | 1.2 ± 0.25 | 1.3 ± 0.15 | 1.0 ± 0.06 | 1.3 ± 0.21 |
| CD4–8+TCRαβ$^{low}$ | 2.2 ± 0.14 | 2.3 ± 0.16$^{**b}$ | 2.0 ± 0.01 | 1.5 ± 0.05$^{*a}$ |
| CD4+8+TCRαβ$^{low}$ | 39.0 ± 0.58 | 37.4 ± 0.79$^{*b}$ | 38.2 ± 0.62 | 39.7 ± 0.15$^{*a}$ |
| CD4–8-TCRαβ$^{high}$ | 0.4 ± 0.08$^{**c}$ | 0.3 ± 0.06$^{**b}$ | 0.6 ± 0.01 | 0.6 ± 0.03 |
| CD4+8-TCRαβ$^{high}$ | 7.1 ± 0.20 | 7.1 ± 0.50 | 7.1 ± 0.01 | 6.8 ± 0.07$^{*a}$ |
| CD4–8+TCRαβ$^{high}$ | 3.4 ± 0.14$^{*c}$ | 3.0 ± 0.30$^{**b}$ | 2.8 ± 0.01 | 2.2 ± 0.03$^{**a}$ |
| CD4+8+TCRαβ$^{high}$ | 6.6 ± 0.54 | 8.0 ± 0.63$^{**b}$ | 7.2 ± 0.09 | 5.8 ± 0.21$^{**a}$ |

The results arc expressed as mean ± SEM (n=5–7).

$^*$ p < 0.05; $^{**}$ p ≤ 0.01

[a] OVX vs. Sham-OVX.

[b] ORX vs. OVX.

[c] Sham-ORX vs. Sham-OVX.

There were significant differences in the relative proportion of none of TCR$\alpha\beta^{low}$ subsets of thymocytes between male and female sham-gonadectomized rats. Orchidectomy also affected the relative proportion of none of subsets of TCR$\alpha\beta^{low}$ cells, while ovariectomy evoked a significant ($p<0.01$) decrease in the percentage of CD4–8+TCR$\alpha\beta^{low}$ cells, and increased the relative proportion of CD4+8+TCR$\alpha\beta^{low}$ cells, thus evoking gender-specific differences in the composition of TCR$\alpha\beta^{low}$ thymocyte subsets. Namely, in gonadectomized rats, the relative proportion of CD4–8+TCR$\alpha\beta^{low}$ cells was significantly ($p<0.01$) higher and that of CD4+8+TCR$\alpha\beta^{low}$ thymocytes significantly ($p<0.05$) lower in males than in females (Table IV).

The relative proportion of CD4–8-TCR$\alpha\beta^{high}$ thymocytes was significantly ($p<0.01$) lower, while that of CD4–8+TCR$\alpha\beta^{high}$ cells was significantly ($p<0.05$) higher in male than in female sham-gonadectomized rats. Orchidectomy had impact on the relative proportion of none of thymocyte TCR$\alpha\beta^{high}$ subsets. However, the ovariectomy significantly decreased the percentage of CD4+8+TCR$\alpha\beta^{high}$, as well as the relative proportions of cells belonging to both subsets of SP cells expressing TCR$\alpha\beta$ at high density (CD4+8-TCR$\alpha\beta^{high}$, CD4–8+TCR$\alpha\beta^{high}$ cells). Therefore, in gonadectomized rats the relative proportions of CD4+8+TCR$\alpha\beta^{high}$ and CD4–8+TCR$\alpha\beta^{high}$ thymocytes were significantly ($p<0.01$) higher, while the percentage of CD4–8-TCR$\alpha\beta^{high}$ cells was significantly ($p<0.01$) lower in male compared with female rats (Table IV).

### DISCUSSION

The present study demonstrated that: a) in spite of age both the thymus size and cellularity were greater in male than in female controls; b) in 2-month-old rats both orchidectomy and ovariectomy increased the thymus size and cellularity, but ovariectomy was more effective in this respect; c) in rats aged 6 months at time of surgery only ovariectomy evoked this effect (orchidectomy was ineffective in this respect), but it was less effective than in younger animals; d) sexual dimorphism in the thymus size and cellularity remained after gonadectomy in rats of both ages; in rats two months old at time of gonadectomy both the thymus weight and cellularity were greater in males than in females, and vice versa in older rats.

The data showing that, in spite of age, both the absolute thymus weight and cellularity were greater in male than in female adult rats are in keeping with our previous observations (Leposavić et al., 1996) and data showing in three strains of rat that after four weeks of age the thymus grows faster in male than in female rats of the same strain (Bellamy et al., 1976). In both adult male and female rats, gonadectomy-evoked increase in the thymus weight and cellularity has also been already demonstrated (Grossman, 1985; Fitzpatrick et al., 1985; Utsuyama and Hirokawa, 1989; Olsen et al., 1991, 1998; Fukumoto, 1997). In favour of the present finding that ovariectomy is more effective than orchidectomy in inducing the thymus enlargement are data: a) showing that, compared with androgens, estradiol is more potent as thymolytic agent (Kumar et al., 1995), and b) suggesting that mechanisms by which estrogens produce the thymus involution are different from that involved in the androgen-induced organ involution (Gulino et al., 1985; Olsen et al., 1994; Kumar et al., 1995; Forsberg, 1996).

The increase in the thymus cellularity, and hence its size, in gonadectomized rats of both sexes may be achieved by: a) enhanced immigration of the thymocyte precursors; b) accelerated thymocyte proliferation; c) decreased thymocyte apoptosis and d) reduced egress of the thymocytes. It has been published that the thymus enlargement in the mice with androgen level lowered by castration occurs owing largely to an accelerated rate of thymocyte proliferation (Olsen et al., 1994). It has also been reported that a lack of estrogen-induced antiproliferative effect is associated with the thymus enlargement evoked by ovariectomy (Forsberg, 1996). However, it has been hypothesized that the mechanisms of antiproliferative action of androgens on thymocytes differ from those of estrogens (Olsen et al., 1994; Gulino et al., 1985). In support to this assumption are data demonstrating that mechanisms of androgen-induced suppression of thymocyte proliferation include enhanced production of TGF-β by TEC (Olsen et al., 1994), while estrogens, in addition to indirect (via modulation of TEC secretory activity) exert a direct antiproliferative effect on the thymus lymphoid cells (Gulino et al., 1985; Forsberg, 1996). Additionally, in male rats, a decreased rate of thymocyte apoptosis (most likely, due to increased expression of Bcl2 protein) as a mechanism of gonadectomy-induced thymus enlargement has been recognized (Olsen et al., 1998). On the other side, direct demonstration of estrogen-mediated thymocyte apoptosis has not been reported, yet. Finally, it appears that the alterations in thymocyte trafficking which may contribute to the regulation of thymus size are more prominent for estrogens than for androgens (Kovacs and Olsen, 1998). In keeping with this observation are data that estrogen administration causes an increase in thymus vascular permeability by direct effect on vascular endothelia (Martin et al., 1995).

Although the age-related change in sensitivity to effects of gonadectomy on the thymus size has been already demonstrated (Utsuyama et al., 1995), the lack of any effect of gonadectomy on the thymus size and cellularity in male rats 6-month-old at time of surgery was surprising. This discrepancy between the results hereby presented and some previously published (Greenstein et al., 1986) may be related to the following facts: a) rats used in the previous study were of different strain (Wistar CSE strain) and age (old between 12 and 15 months) and b) the relative (expressed as mg thymic tissue / 100 g body weight) but not absolute weights of their thymi were compared. The lack of effects on the thymus cellularity might be related to data suggesting that the age-related alterations in thymus sensitivity to gonadal deprivation mainly reflect the changes in the thymus microenvironment (Utsuyama et al., 1995).

The apparent sexual dimorphism in age-related decline of thymus sensitivity to action of gonadal hormones might be explained by gender-specific differences in age-related reduction in ($H^3$) thymidine incorporation into thymocytes (Segal et al., 1985). In rats of both sexes ($H^3$) thymidine incorporation into thymocytes is at its maximum at the second month of age, and thereafter, in males, the incorporation of this marker declines rapidly; whereas in females, the decline starts only after 6 months of age (Segal et al., 1985). Namely, the sensitivity of thymocytes to ConA stimulation do not significantly differ between male and female rats, but since the thymocyte basal proliferation rate decreases with age in gender-specific way, the blastogenic response of thymocytes to ConA stimulation also exhibits age-related sexually dimorphic decline (Segal et al, 1985). Therefore, differential effects of gonadectomy in rats of different sex at distinct age points might be associated with age-related sexually dimorphic changes in the proliferative capacity of thymocytes.

The results also revealed that, regardless of sex, in 2-month-old rats at time of surgery the increase in volumes of both the cortex and medulla contributed to the thymus enlargement, but that the importance of the first was quantitatively greater. These data are in agreement with the previous observations that changes in the number of both cortical and medullary thymocytes contribute to thymus involution evoked by estradiol treatment, but that contribution of the first is quantitatively more significant (Martin et al., 1994). The disproportional contribution of two main thymus compartments in the organ enlargement was more conspicuous in female than in male rats, that further supports the notion that gender-specific mechanisms are involved in the gonadectomy-evoked thymus enlargement. In rats OVX at age of 6 months, the increase in the volume of thymus cortex was also greater than that of medulla; this increase was less pronounced than that in younger animals, while the increase in volume of medulla did not differ from that in younger rats. Therefore, it seems that the age-related decrease in the sensitivity of the whole organ to ovariectomy mainly reflected a decline in the cortex sensitivity to this treatment.

Moreover, the present results showed that in OVX rats of both ages the increase in medullary volume was more remarkable than that in the number of medullary thymocytes, and that this effect was even more conspicuous in older rats. These findings suggest that: a) in the increase in the volume of thymus medulla, besides lymphoid, participated also the non-lymphoid cell component and b) the contribution of this component was greater in six-month-old rats compared to younger animals. These data are in agreement with the previous reports which emphasize the importance of non-lymphoid cell component in the estrogen-induced thymus involution (Clark and Kendall, 1989; Martin et al., 1994), as well as with those indicating that, in female rats, the surgical ablation of gonads causes an increase in the number of thymulin-containing TEC (Dardenne et al., 1988), which are shown to contain the receptors for both estrogens and progesterone (Fujii-Hanamoto et al., 1985; Kawashima et al., 1991). Contrary to rats OVX at age of two months, in rats ORX at the same age, the increase in the volume of medulla was less conspicuous than that in the number of medullary thymocytes suggesting that, in these rats, the volume of non-lymphoid cell component was either decreased or that the volume of this component remained unaltered. These findings are compatible with data showing that: a) TEC contain the receptors for androgens and b) androgens administration to TEC culture generates a biphasic proliferative pattern, whereby higher concentrations than those required for maximal cell yield result in dose-dependent inhibitory effect (Sakabe et al., 1994).

The phenotypic analysis of thymocytes showed that the composition of thymocyte subsets significantly differed between sham-operated male and female rats of both ages, as well as that the differences in thymocyte composition between these rats were age-dependent. These findings are in agreement with our previous report (Leposavić et al., 1996). Both orchidectomy and ovariectomy had impact on the thymocyte composition in rats aged two months at time of surgery, while in older rats only ovariectomy evoked changes. In younger rats, the effects evoked by orchidectomy differed from that induced by ovariectomy, that is also in agreement with some previous findings (Leposavić et al., 1996; Kovacs and Olsen, 1998). Finally, sexual dimorphism in the thymocyte composition was demonstrated in gonadectomized rats, as well.

In sham-operated rats of both age the relative proportion of CD4–8-TCRαβ$^{high}$ thymocytes belonging to a subset of mature cells which harbors potentially autoreactive clones (Budd and Mixter, 1995) was lower in male compared with female rats. These findings might be associated with well-known lower incidence of autoimmune diseases in males compared with females (Roubinian et al., 1977a, 1978, 1979; Steinberg et al., 1980; Pozzilli et al., 1993; Vidal et al., 1994). In male sham-ORX rats of both ages the relative proportion of the mature CD4–8+TCRαβ$^{high}$ thymocytes was higher. In male rats sham-orchidectomized at age of two months the relative proportion of the another subset of the most mature SP thymocytes (CD4+8-TCRαβ$^{high}$) was also higher. In these rats, differences in the relative proportions of thymocytes expressing undetectable or low levels of TCRαβ, were observed, as well. However, the significance of these findings for the immunological sexual dimorphism, and especially for higher propensity of autoimmune diseases in females, is difficult to explain at present.

Ovariectomy in two-month-old rats evoked a decrease in the relative proportion of the least mature CD4–8-TCRαβ$^{-}$, that is consistent with the proposed role for estrogens in the regulation of immigration of the bone marrow-derived precursor cells into the thymus (Forsberg, 1996). Orchidectomy, in the rats of the same age increased the percentages of CD4–8+TCRαβ$^{low}$ and CD4+8+TCRαβ$^{low}$ cells and decreased the relative proportions of both subsets of SP cells expressing TCRαβ at high level, that is in agreement with the previous reports (Olsen et al., 1994). The increased percentage of CD4+8+ TCRαβ$^{low}$ cells might reflect either a decreased rate of thymocyte apoptosis and/or a decreased rate of thymocyte transition from this stage to the stage of CD4+8+TCRαβ$^{high}$ which requires the process of positive selection to be passed (Jameson et al., 1995). Since the relative proportion of CD4+8+TCRαβ$^{high}$ cells remained unaltered, while the relative proportions of both subsets of the SP mature cells were decreased, the first option would suggest an increased egress of mature thymocytes into periphery, while the second one would implicate a more efficient differentiation of the CD4+8+TCRαβ$^{high}$ cells which passed positive selection (Jameson et al., 1995) into the mature cells of both CD4+8-TCRαβ$^{high}$ and CD4–8+TCRαβ$^{high}$ phenotypes, as well. The former option is fully consistent with the reduced thymocyte trafficking in male rats due to the presence of androgens (Kovacs and Olsen, 1998).

Ovariectomy in rats aged 6 months decreased the relative proportion of CD4–8+TCRαβ⁻ and that of CD4–8+TCRαβ$^{low}$ thymocytes. The cells of these phenotypes are shown to be a direct precursors of DP cells in both rats (Hunig et al., 1989) and mice (Tsuchida et al., 1994). The reduced percentage of these cells, together with that of CD4–8-TCRαβ$^-$, is consistent with already mentioned role of estrogens in the regulation of the thymocyte precursor immigration (Fosberg, 1994). However, if we take into consideration that, in these rats, the relative proportions of both CD4+8+TCRαβ$^-$ and CD4+8+TCRαβ$^{low}$ cells were increased, the decreased percentages of CD4–8-TCRαβ$^-$, and both CD4–8+TCRαβ$^-$ and CD4–8+TCRαβ$^{low}$ thymocytes might be related to an accelerated transition of these cells to the stage of DP cells. The increase in the relative proportions of both CD4+8+TCRαβ$^-$ and CD4+8+TCRαβ$^{low}$, together with the decrease in relative proportion of CD4+8+TCRαβ$^{high}$ thymocytes, and reduction in percentage of the cells into which this subpopulation differentiates (i.e. CD4+8-TCRαβ$^{high}$ and CD4–8+TCRαβ$^{high}$), is fully in agreement with the previous finding showing that estrogens *in vitro* accelerate thymocyte positive selection, as well as differentiation of positively selected cells into both subsets of mature cells (Screpanti et al., 1989).

In conclusion, the gonadectomy in adult rats evoked sex- and age-dependent effects on thymus size and cellularity, as well as on the composition of twelve thymocyte subsets delineated by expression of CD4, CD8 molecules and TCRαβ, that clearly indicate sex-dependent changes in the modulatory role of gonadal hormones in regulation of T-cell maturation, and possibly immune response. In addition, from the present results it is also apparent that gonadectomy, in adult rats, influences, but not abolishes, the sexual dimorphism in thymus size and cellularity, as well as that in thymocyte subsets composition.

### MATERIALS AND METHODS

### Animals

Inbred AO rats of both sexes aged 2 and 6 months at the beginning of experiment were used. Animals were kept under routine laboratory conditions and provided with food and water ad libitum.

### Experimental Protocol

Rats aged 2 and 6 months were bilaterally gonadectomized or sham-gonadectomized under sodium pentobarbitone anesthesia (Nembutal, Serva, 40 mg/kg, i.p.). Orchidectomy was performed through the scrotal incision. In sham-operated males, testes were identified but not removed, and the incision was closed. Females were ovariectomized through dorso-lateral incisions. Rats subjected to complete surgical procedure except removing the ovaries were used as sham-operated controls. One month after surgery, the rats were killed, their thymuses removed out carefully, freed from extraneous tissue, weighed and appropriately processed for subsequent stereological or flow cytometric analysis (FCA).

### Stereological analysis

The left thymus lobes were fixed in Bouin's solution, embedded in paraffin and sectioned transversely on a Reichert microtome, at a section thickness of 5 μm. The sections were stained with haematoxylin and eosin and analyzed under a Reichert microscope equipped with an eyepiece fitted with Weibel' s M42 test system. The test area was always chosen at random, and the size of the final sample was calculated separately for each parameter to provide results within the confidence interval of 95% (Karapetrović et al., 1995).

Absolute volumes of the thymus cortex and medulla were estimated from the absolute volume of the organ and volume density (Vv) of appropriate compartment. The absolute volume of thymus was calculated by dividing the thymus weight by 1.05 g/cm$^3$, as has been suggested by Casley-Smith (1988). Vv (represents part of tissue volume occupied by the analyzed structure) of the thymus cortex and medulla were determined under a magnification × 6.3, using a point counting method. They were calculated according to the equation: $Vv = Pf/Pt$, where

Pf = the number of test points falling on the analyzed structure and Pt = the total number of test points (42 for the M42 test system).

The number of thymocytes in the cortex was calculated from the numerical density (Nv) of cortical thymocytes and absolute volume of thymus cortex taking into consideration that three quarters of the cortical thymocytes are situated within the deep cortex (Karapetrović et al., 1995). The number of the thymocytes located in the medulla was calculated from their Nv (represents number of thymocytes per volume unit of appropriate thymus tissue) and the absolute volume of thymus medulla. The Nv of thymocytes was estimated under immersion magnification. The test lattice was placed randomly, but positioned parallel to, and just touching, the capsule (for the outer cortex analysis) and the cortico-medullary junction (for the deep cortex analysis). For estimating Nv of medullary thymocytes the lattice was placed randomly throughout the medulla. Nv of thymocytes was calculated from the equation: $Nv = NA/(\bar{D} + Go)$, where NA stands for number of thymocytes per surface unit of test area, and was estimated from the relation: NA=N/At (N=number of thymocytes per test area; At=size of test area); $\bar{D}$ for the mean caliper diameter of thymocytes and was calculated according to the following equation: $\bar{D} = 6Vv/Sv$, where Sv=2If/Lt (If=the number of intersections of the test lines with the plasma membrane of the thymocytes and Lt=the total length of test lines) and Go for depth sharpness and was determined from the equation: Go=1/(n+Na) (1=wave length of light; n=coefficient of the immersion oil diffraction; Na=numerical aperture of the objective lens).

### Flow Cytometry

### *Preparation of thymocyte single-cell suspension*

Thymocyte suspensions were prepared by passing the thymus tissue through the stainless steel mesh into ice-cold PBS (pH 7.3) containing 2% fetal calf serum (Gibco, Grand Island, NY, USA) and 0.01% sodium azide (PS medium). After washing three times in PS medium, the single-cell suspensions were counted in a standard haemocytometer, adjusting the cell concentration to $1 \times 10^7$ cells/ml. The viability of such cell preparations, as determined by Trypan blue exclusion, was routinely greater than 95%.

### *Three-color immunofluorescence staining of thymocyte CD4, CD8 and TCRαβ*

The aliquots of $1 \times 10^6$ thymocytes were incubated for 30 min at 4°C, in the dark, with the following antibodies: fluorescein-isothiocyanate (FITC)-conjugated anti-CD4 (clone W3/25, Serotec, Oxford, UK); phycoerythrin (PE)-conjugated anti-CD8 (clone MRC OX-8, Serotec) and biotin-conjugated anti-TCRαβ (clone R73, Serotec). After incubation, the aliquots were washed three times in PS medium, and incubated for the next 30 min at 4°C, in the dark, with the second-step reagent streptavidin peridinin chlorophill protein (PerCP) (Becton Dickinson, Mountain View, CA, USA). Following three washes in PS medium, the cells were fixed in 0.5 ml 1% paraformaldehyde and kept at 4° C in the dark until analysis. All samples were analyzed on the same day on a FACScan flow cytometer (Becton Dickinson). Dead cells were routinely excluded on the basis of forward light scatter and side light scatter. Usually $5 \times 10^4$ flow cytometric events were analyzed. The analyses were carried out with respect to appropriate isotypic and fluorochrome-matched controls, with FACScan Research Software program (Becton Dickinson).

### Statistical Analysis

The data were expressed as mean ± SEM. One-way analysis of variance (ANOVA) followed by LSD test for post hoc comparisons was performed for determining the differences between means, using the SPSS 7.5 for Windows software package and IBM compatible PC.

### *References*

Agnello V., Pariser K., Gell J., Gelfrand J., and Turksoy R.N. (1983). Preliminary observations on danazol therapy of systemic lupus erythematosus: effects on DNA antibodies, thrombocytopenia and complement. J Rheumatol 10:682–687.

Ansar-Ahmed S., Penhale W.J., and Talal N. (1985). Sex hormones, immune responses, and autoimmune diseases. Mechanism of sex hormone action. Am J Pathol 121:531–551.

Bellamy D, Hinsull SM., and Phillips JG. (1976). Factor controling growth and age involution of the rats thymus. Age Ageing 5:12–19.

Ben-Chetrit A., and Ben-Chetrit E. (1994). Systemic lupus erythematosus induced by ovulation induction treatment. Arthritis Rheum 37:1614–1617.

Bizzaro A., Valentini G., Di Martino G., Daponte A., De Bellis A., and Iacono G. (1987). Influence of testosterone therapy on clinical and immunological features of autoimmune diseases associated with Klinefelter's syndrome. J Clin Endocrinol Metab 64:32–36.

Budd R.C., Mixter P.F. (1995). The origin of CD4-CD8-TCRαβ+ thymocytes: a model based on T-cell receptor avidity. Immunol Today 16:428–431.

Casley-Smith J.R. (1988). Expressing stereological results "per $cm^3$" is not enough. J Pathol 156:263–265.

Clarke A.G., and Kendall M.D. (1989). Histological changes in the thymus during pregnancy. The thymus in pregnancy Thymus 14:65–78.

Fitzpatrick F.T., Kendall M.D., Wheeler M.J., Adcock I.M., and Greenstein B.D. (1985). Reappearance of thymus of aging rats after orchidectomy. J Endocrinol 106: R17-R19.

Forsberg J.-G. (1996). The different responses of the female mouse thymus to estrogen after treatment of neonatal, prepubertal, and adult animals. Acta Anat 157: 275–290.

Fujii-Hanamoto H, Seiki K, Sakabe K, and Ogawa H. (1985). Progestin receptor in the thymus of ovariectomized immature rats. J Endocrinol 107: 223–229.

Fukumoto T. (1997). Precise analyses of the structure of the thymus for establishing details of the mechanisms underlying thymocyte proliferation and maturation. Arch Histol Cytol 60:1–8.

Graff R.J., Lappe M.A., and Snell G.D. (1969). The influence of the gonads and adrenal glands on the immune response to skin grafts. Transplantation 7:105–111.

Greenstein B.D., Fitzpatrick, F.T.A., Adcock I.M., Kendall M.D., and Wheeler M.J. (1986). Reappearance of the thymus in old rats after orchidectomy: inhibition of regeneration by testosterone. J Endocrinol 110:417–422.

Grossman C.J., Nathan P., and Taylor B.B. (1979). Rat thymic dihydrotestosterone receptor: preparation, localization and physicochemical properties. Steroids 34: 539–553.

Grossman C.J. (1984). Regulation of the immune system by sex steroids. Endocr Rev 5: 435–455.

Grossman C.J. (1985). Interactions between the gonadal steroids and the immune system. Science 227:257–261.

Grossman C.J. (1990). Are there underlying immune-neuroendocrine interactions responsible for immunological sexual dimorphism. Prog Neuroendocrimmunol 3:75–82.

Gulino A., Screpanti I., Torrisi M.R., and Frati L. (1985). Estrogen receptors and estrogen sensitivity of fetal thymocytes are restricted to blast lymphoid cells. Endocrinology 117:47–54.

Hunig T., Wallny H-J., Hartley J.K., Lawetzky A., Tiefenthaler G. (1989). A monoclonal antibody to a constant determinant of the rat T cell antigen receptor that induces T cell activation. J Exp Med 169:73–86.

Jameson S.C., Hogquist K.A., and Bevan M. (1995). Positive selection of thymocytes. Ann Rev Immunol 13:93–126.

Karapetrović, B., Mićić, M., and Leposavić, G. (1995). Stereological analysis of the sexually mature rat thymus after orchidectomy. Indian J Med Res 102:42–48.

Kawashima I., Sakabe K., Seiki K., Fujii-Hanamoto H., Akatsuka A., and Tsukamoto H. (1991). Localization of sex steroid receptor cells, with special reference to thymulin (FTS)-producing cells in female rat thymus. Thymus 18:79–93.

Kawashima I., Seiki K., Sakabe K., Ihara S., Akatsuka A., and Katsumata Y. (1992). Localization of estrogen receptors and estrogen receptor-mRNA in female mouse thymus. Thymus 20:115–121.

Kohen F., Abel L., Sharp A., Amir-Zaltsman Y., Somjen D., Luria S., Mor G., Knyszynski A., Thole H., and Globerson A. (1998). Estrogen-receptor expression and function in thymocytes in relation to gender and age. Dev Immunol 5:277–285.

Kovacs WJ., and Olsen N.J. (1998). Sex hormones and immune responses. In Contemporary Endocrinology: Autoimmune Endocrinopathies, Volpe R., Ed. (Totowa, NJ: Humana Press Inc.), pp 163–181.

Kumar N., Shan L-X., Hardy M.P., Bardin C.W., and Sundaram K. (1995). Mechanism of androgen-induced thymolysis in rats. Endocrinology 136:4887–4893.

Leposavić G., Karapetrović B., Budeč M., and Kosec D. (1995). Sex differences in the phenotypic characteristics of rat thymocytes. Acta Vet 45:215–220.

Leposavić G., Karapetrović B., Obradović S., Vidic-Danković B., and Kosec D. (1996). Differential effects of gonadectomy on the thymocyte phenotypic profile in male and female rats. Pharmacol Biochem Behav 54:269–276.

Martin A., Alonso L., Gómez del Moral M., and Zapata A.G. (1994). Morphometrical changes in the rat thymic lymphoid cells after treatment with two different doses of estradiol benzoate. Histol Histopath 9:281–286.

Martin A., Casares F., Alonso L., Nieuwenhuis P., Vincente A., and Zapata A.G. (1995). Changes in the blood thymus barrier of adult rats after estradiol treatment. Immunobiology 192:231–248.

Olsen N.J., Watson M.B., Henderson G.S., and Kovacs W.J. (1991). Androgen deprivation induces phenotypic and functional changes in the thymus of adult male mice. Endocrinology 129:2471–2476.

Olsen N.J., Viselli S., Shults K., Stelzer G., Kovacs W.J. (1994). Induction of immature thymocyte proliferation after castration of normal male mice. Endocrinology 134:107–113.

Olsen N.J., Viselli S.M., Fan J., and Kovacs W.J. (1998). Androgens accelerate thymocyte apoptosis. Endocrinology 139:748–752.

Pozzilli P., Signore A., Williams A.J., and Beales P.E. (1993). NOD mouse colonies around the world-recent facts and figures. (Review). Immunol Today 14:193–196.

Petrie H.T., Hugo P., Scollay R., and Shortman K. (1990). Lineage relationships and developmental kinetics of immature thymocytes: CD3, CD4, and CD8 acquisition *in vivo* and *in vitro.* J Exp Med 172:1583–1588.

Quaglino D., Capri M., Bergamini G., Euclidi E., Zecca L., Franceschi C., and Ronchetti I.P. (1998). Age-dependent remodeling of rat thymus. Morphological and cytofluorimetric analysis from birth up to one year of age. Eur J Cell Biol 76:156–166.

Roubinian J.R., Papoian R., and Talal N. (1977a). Androgenic hormones modulate autoantibody responses and improve survival in murine lupus. J Clin Invest 59:1066–1070.

Roubinian J.R., Papoian R., and Talal N. (1977b). Effects of neonatal thymectomy and splenectomy on survival and regulation of autoantibody formation in NZB/NZW F1 mice. J Immunol 118:1524–1529.

Roubinian J.R., Talal N., Greenspan J.S., Goodman J.R., and Siiteri P.K. (1978). Effects of castration and sex hormone treatment on survival, anti-nucleic acid antibodies, and glomerulonephritis in NZB/NZW F1 mice. J Exp Med 147:1568–1583.

Roubinian J.R., Talal N., Greenspan J.S., Goodman J.R., and Siiteri P.K. (1979). Delayed androgen treatment prolongs survival in murine lupus. J Clin Invest 63:902–911.

Sakabe K., Seiki K., Kawashima I., Katsumata Y., Itoh T. (1992). An effect of steroid hormones on proliferation and function of thymic epithelial cells in culture. Rec Adv Cell Molec Bio 1:141–150.

Scollay R., Bartlett P., and Shortman K. (1984). T cell development in the adult thymus: Changes in the expression of the surface antigens Ly2, L3T4 during development from early precursor cell to emigrants. Immunol Rev 82:79–103.

Screpanti I., Morrone S., Meco D., Santoni A., Gulino A., Paolini R., Crisanti A., Mathieson B.J., and Frati L. (1989). Steroid sensitivity of thymocyte subpopulations during intrathymic differentiation. Effects of 17β-estradiol and dexamethasone on subsets expressing T cell antigen receptor or IL-2 receptor. J Immunol 142: 3378–3383.

Segal J., Troen B.R., and Ingbar S.H. (1985). Effects of age and sex on certain metabolic functions and mitogenic activity in rat thymocytes. Thymus 7:211–220.

Seiki K., Sakabe K., Fujii-Hanamoto H., Kawashima I., Ogawa H., Akatsuka A., and Yanaihara N. (1988). Localization of sex steroid receptor cells in the thymus, with special reference to thymic factor-producing cells. Med Sci Res 16:967–970.

Steinberg A.D., Roths J.B., Murphy E.D., Steinberg R.T., and Raveche E.S. (1980). Effects of thymectomy or androgen administration upon the autoimmune disease of MRL/Mp-lpr/lpr mice. J Immunol 125:871–873.

Tsuchida M., Konishi M., Jojima K., Naito K., Fujikura Y., and Fukumoto T. (1994). Analysis of cell surface antigens on glucocorticoid-treated rat thymocytes with monoclonal antibodies. Immunol Letters 39:209–217.

Utsuyama M., Hirokawa K., Mancini C., Brunelli R., Leter G., and Doria G. (1995). Differential effects of gonadectomy on thymic stromal cells in promoting T cell differentiation in mice. Mech Age Dev 81:107–117.

Utsuyama M., and Hirokawa K. (1989). Hypetrophy of the thymus and restoration of immune functions in mice and rats by gonadectomy. Mech Age Dev 47:175–185.

Vidal S., Gelpi C., and Rodriguez-Sanchez J.L. (1994). (SWR × SJL)F1 mice: a new model of lupus-like disease. J Exp Med 179:1429–1435.

Viselli S.M., Olsen N.J., Shults K., Stcizer G., and Kovacs W.J. (1995). Immunochemical and flow cytometric analysis of androgen receptor expression in thymocytes. Mol Cell Endocrinol 109:19–26.

